# The Caregiving Health Engagement Scale (CHE-s): development and initial validation of a new questionnaire for measuring family caregiver engagement in healthcare

**DOI:** 10.1186/s12889-019-7743-8

**Published:** 2019-11-27

**Authors:** Serena Barello, Cinzia Castiglioni, Andrea Bonanomi, Guendalina Graffigna

**Affiliations:** 1 EngageMinds Hub – Consumer & Health Research Center, Department of Psychology, Università Cattolica del Sacro Cuore, L.go Gemelli 1, 20123 Milan, Italy; 2 Department of Psychology, Università Cattolica del Sacro Cuore, Milan, Italy; 3 Department of Statistical Science, Università Cattolica del Sacro Cuore, Milan, Italy; 4 EngageMinds Hub – Consumer & Health Research Center, Department of Psychology Faculty of Agricultural, Nutrition and Environmental Sciences -Università Cattolica del Sacro Cuore, Milan, Italy

**Keywords:** Family caregiver, engagement, Psychometric scale, Scale validation, Questionnaire, healthcare, family engagement, Chronic care, Psychology, CHE-s

## Abstract

**Background:**

This study was aimed to preliminary validate a cross-disease psychometric measure to assess the psycho-social experience of family caregiver engagement in healthcare (Caregiving Health Engagement Scale, CHE-s), which refers to the caregiver’s psychological attitude to be an active, skilled and motivated player in the care process of their loved ones.

**Method:**

The study consisted of a mixed methods, multi-stage research. First, a preliminary qualitative stage was aimed at investigating – in the caregivers’ perspective - the engagement process in providing care to a ill relative (stage 1). The second stage of the research was aimed at developing a psychometric scale to assess this concept (i.e. the Caregiving Health Engagement Scale – CHE-s) and to preliminary test its psychometric properties (stage 2).

**Results:**

Overall, 230 caregivers (32 in stage 1, and 198 in stage 2) participated to the study. The first qualitative stage, conducted by qualitative interviews on 32 family caregivers, highlighted four main experiential positions of caregiver engagement (namely, *denial*, *hyper-activation*, *drawing*, and *balance*), showing that “full engagement” occurs when caregivers become able to *reach balance* between their caring tasks and their broad life goals. In the second quantitative stage, we used the qualitative evidences emerged from stage one as a basis for developing the items of the Caregiving Health Engagement scale (CHE-s). We preliminary tested its psychometric properties through a cross-sectional study on 198 caregivers, which demonstrated CHE-s to be a reliable measure to capture the dynamic nature of caregiver engagement. The CATPCA results, together with the ordinal alpha of 0.88, suggests a mono-dimensional latent structure and a very good internal consistency and CFA showed adequate goodness of fit indices. (CFI = 0.96, RMR = 0.03, RMSEA = 0.05).

**Conclusions:**

Health care systems that prioritize person-led care may benefit from using the Caregiving Health Engagement Model and the CHE scale (CHE-s) to assess the engagement level of family caregivers in order to better tailor the supportive and educational intervention addressing them.

## Background

### Who are family caregivers in the contemporary healthcare?

Worldwide population is aging and the demand of care is growing more and more. To address this societal challenge, the role of family caregivers is becoming increasingly important in providing support alongside the formal healthcare services [1, 2]. While healthcare is most commonly understood as medical services provided by health care professionals, not all the forms of care services are provided formally. In fact, the most of care is provided informally (by family members of friends) than formally.

A recent report of the European Commission [3] showed that approximately 60% of the 20.7 million dependent elderly in the EU received informal care, thereby highlighting that informal caregivers remain the most important group of providers [in the EU]. For the United States, 39.6 million people in the American population aged 15 and over are engaged in elderly care provision [4].

According to the definition offered by the Family Caregiver Alliance [5–7], a family caregiver is a person who provides continuous care and assistance, without being paid, for a family member who is in need of support due to physical, cognitive or mental health conditions. A family caregiver is, in other words, someone who is informally responsible for the physical, emotional and often financial support of another person who is unable to care for him/herself due to illness, injury or disability. The care recipient may be a family member, life partner or friend. For these reasons, family caregivers are recognized as the backbone of the health care system and supporting them has become an international public health priority given their essential role [8].

This essential role implies that healthcare providers start to consider family caregivers as active and direct partners in the healthcare journey in order to benefit from their support [9]. Indeed, the willingness of individuals to act and be engaged as carers of their loved ones is crucial to sustain informal care resources as part of the health care provision.

Engaging family caregivers in the healthcare process of their loved ones is currently recognized to be a key pillar for improving services’ effectiveness and sustainability [10–15]. However, caregivers are largely unrecognized as members of the health care team [16, 17]. Identifying effective ways to implement patient and family engagement is fundamental to improve the patient and family experience as well as to improve safety, quality, and delivery of care.

### Why does engaging family caregivers matter?

Many are the functions and roles of family caregivers such as advocating for the healthcare rights of the patient, supporting patients during the medical consultation [18, 19], supporting the hospital discharge process as well as supporting the patients in attending follow ups and controls [20–23]. Finally nowadays family caregivers are an important linkage among the different services and healthcare professionals, and they help achieving the goal of a real integrated model of care [22, 24, 25]. Caregiver engagement is especially crucial in cases that involve medically frail patients such as the elderly, people affected by mental disorders or neurodegenerative diseases, and children [26–29]. Some scholars have demonstrated how the therapeutic alliance and communication with providers improve when family caregivers participate in the medical consultation: when effectively engaged, family caregivers ask doctors more questions and are more effective information seekers in comparison to their assisted patient [30]. Furthermore, family caregivers might enhance patients’ adherence to treatment [31, 32]. Finally, family caregivers are often the patients’ main source of psycho-emotional support [2].

The engagement of family caregivers in supporting patients’ effective health management is such, that some scholars claimed that the caregiving function is a potential surrogate of healthcare [33, 34]. Although this may sound as an extreme position, it appears plausible to say that guaranteeing a synergy and a partnership between family caregivers and the healthcare providers is currently an ethical and pragmatic priority for healthcare systems [35–37]. However, a few studies also pointed out the negative effect that caregivers, if not well supported, may produce in the healthcare relationship: this is the case of family caregivers who bring their own agenda to the medical consultation by excluding or criticizing the patient and by dominating the conversation with the healthcare professional [30, 38, 39]. This is also the case of too protective and too activated caregivers who tend to substitute the patient in his/her decision and basic healthcare function, by passivizing him/her and minimizing his/her possibility to get engaged in his/her healthcare [40].

### The lack of a psycho-social conceptualization of family caregiver engagement

Family caregiver engagement is defined as active partnerships among health care providers, patients, and families [29, 41]. It generally refers to behaviors such as seeking information on the patient’s health condition or sustaining the patient’s participation in making decisions based on the collected information [42]. These decisions primarily involve selecting options on therapeutic regimen and care, but they also include making plans regarding the course of patients’ illness. There are many definitions and conceptualizations of family caregiver engagement in healthcare which emphasize different aspects of the phenomenon [43–45]. For instance, Carman and colleagues define family engagement as a process in which “patients, families, [and] their representatives [are] working in active partnership at various levels across the health care system to improve health and health care” [35]. Brown and colleagues, on the other hand, define caregiver engagement as the active partnership between health professionals and families existing at different levels of the healthcare system, including direct care delivery and care system and service design [41]. Maurer and colleagues, consider family engagement as a “set of behaviors by patients, family members, and health professionals and a set of organizational policies and procedures that foster both the inclusion of patients and family members as active members of the health care team and collaborative partnerships with providers and provider organizations” [46]. These definitions, while differing slightly, underline the behavioral nature of the caregiver engagement as a strategy to promote partnerships among patients, family members, and health care providers at multiple levels, but they often lack of a wider psychosocial understanding of the phenomenon. Similar to the literature on patient engagement [47–50], the study of the caregiver’s subjective experience of being engaged in the process of care (and what actions foster this experience) has been, so far, neglected. It is interesting to note, that the caregiver him/herself is the “great absent” in the discussion about caregiver engagement and there are no studies involving caregivers in defining what engagement means for them.

Indeed, despite the growing consensus about the importance of sustaining the engagement of family caregivers in healthcare [35, 40, 51–53], we could neither find a clear conceptualization of it, nor a validated psycho-metric tool to measure the family caregiver engagement experience rooted in the direct caregivers’ perspective.

Currently, theorizations and psychometrics measures exist to capture only specific aspect of the caring experience such as the stressful consequences of being family caregivers [54–57] and the skills/attitudes/knowledge to actively take part in the loved ones’ care [58, 59]. For instance, a variety of instruments have been developed to measure distressing experiences in terms of caregivers’ burden, needs and the impact of taking care of a relative on the caregivers’ quality of life [55, 60, 61]. Moreover, research has tended to focus on caregiving associated with specific patients’ health conditions and currently available theorizations are mainly focused on specific caregivers population [12, 62–64], thus lacking a wider comprehension of the engagement experience of family caregivers across different disease conditions. A cross-disease measurement of the caregivers’ psycho-social experience of active engagement in the care of their loved ones is still missing. Indeed, understanding the caregiver engagement experience across different illness groups can be useful to optimize support or training dedicated to caregiver on this topic.

From a psycho-social perspective we claim that understanding caregivers’ experiences, attitudes and expectations towards the new “role of carer” is fundamental in order to design supports and initiatives devoted to sustain them in their important caring role. There is, however, a gap between what is known about the caregiver experience and what is most likely to offer support. A better comprehension of the caregiver lived experience and ways to measure their engagement in the care process are needed.

In order to fill the gap found in the literature on this topic, this study was devoted to hear the voices of family caregivers - their struggles, challenges, expectations, and motivation for persevering in their assistance role, as well as their preferences regarding education, resources, and supports that might enhance their engagement.

According to these premises, the aim of the present study was to develop a family caregiver health engagement scale (CHE-s) to measure the caregivers’ psychological sense of being involved in the care of their loved ones. We also tested the CHE-s in a population of the family caregivers.

## Methods

**Fig. 1:**
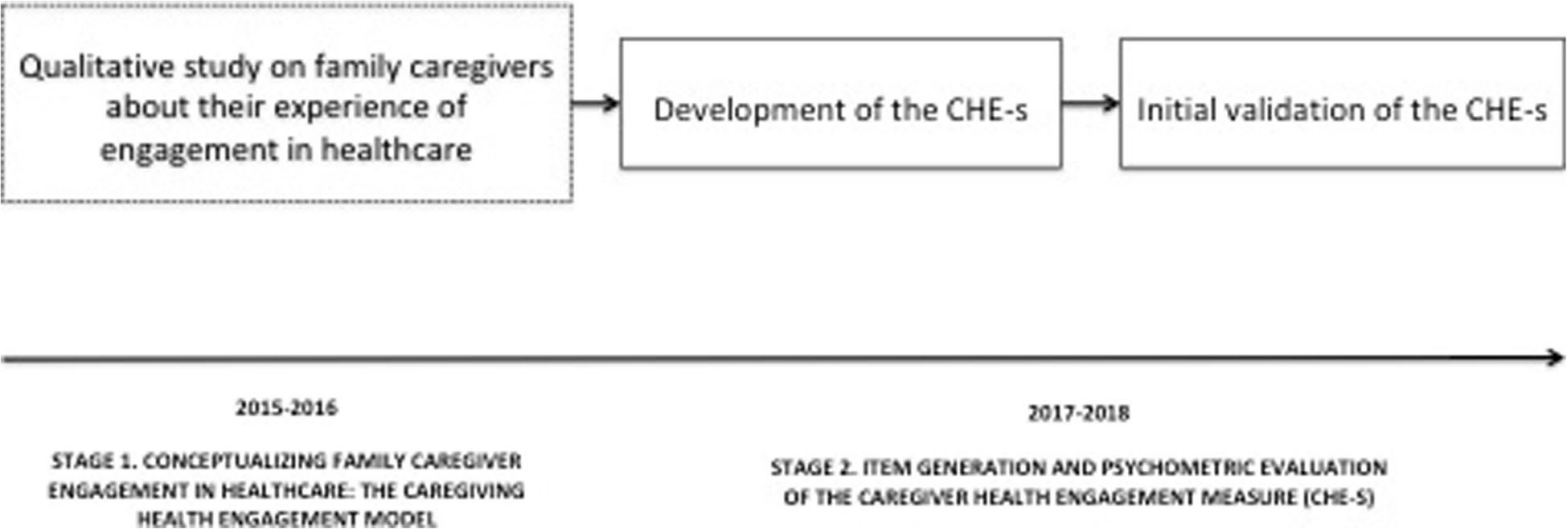
The study design

The development of the family caregiver engagement scale was based on a mixed-method, multi-stage design process. Particularly, the development of the questionnaire and validation study took place in two stages. Stage 1 consisted of the questionnaire development stage, and phase 2 comprised the validation study, which included exploratory and confirmatory analyses (Fig. 1). Following the methodological details of each stage.

### Stage 1. Towards a qualitative conceptualization of family caregiver engagement in healthcare: the caregiving Health engagement model

In stage 1, a qualitative study was conducted to explore what does engagement in healthcare mean to family caregiver of chronic patients, in particular those with complex care needs. To address the research question of this study, this caregiver population is particularly interesting in order to capture the experience of caregiving in the long run and in its multi-faced nature.

#### Sampling

For the aim of qualitative stage of this study, a purposive sample of family caregivers of patients with complex care needs was invited to participate in semi-structured qualitative interviews to examine and discuss their experience of taking care of their loved ones and their views and perspective of their role and engagement in the healthcare process. Inclusion criteria required family caregivers to 1) have been assisting a patient with activities of daily living and complex health care needs for at list 6 months; 2) aged over 18; 3) being a caregiver without cognitive impairment; 4) and able to speak and understand Italian. We followed a maximum variety sample procedure by purposely select caregivers of patients affected by different healthcare conditions and with different level of burden of illness in order to include different experience of caregiving.

#### Recruitment procedure

The participants to the interviews were recruited basing on the advice of Italian general practitioners that suggested to the researchers a list of names that were included or excluded according to the selection criteria. We have tried to recruit caregivers with different characteristics to ensure that individuals from diverse demographic backgrounds and with different experience of caregiving are present in the qualitative stage.

#### Data collection

An “ecological” conceptual model of the psycho-social experience of family caregiver engagement was developed involving qualitative interviews with family caregivers of patients with chronic conditions. Generally, the participants were encouraged to talk about their own feelings and experiences and to discuss the changes they had experienced in their caring roles and feeling of engagement. An interview schedule was developed by SB and GG basing on a literature analysis on this topic but with the aim to guarantee free expression of the participants. Moreover, when developing the interview schedule, the authors were inspired by some their previous qualitative studies conducted on the topic of patient engagement [65–67]. During the interviews, family caregivers were asked to answer the following open questions: *could you describe your feelings as a caregiver for a relative with a chronic condition? Can you give me some examples of activities and roles that you enact or enacted when taking care of your loved one? How a family caregiver becomes actively engaged in the healthcare of his/her loved one? What are the benefits/disadvantages of such engagement? What are the factors in the relationship with the healthcare team that facilitated ore hindered your engagement in the care process?* In addition to these questions, the interviewer added specific probes in order to facilitate the conversation and to deepen the caregivers’ experience (i.e.: this is very interesting, can you deepen this aspect? Can you give me some examples of that?). All caregivers were informed about the aim of the study and their informed consent was obtained. Two researchers (SB and GG) expert in qualitative studies conducted the interviews that lasted on average 52 min. The interviews with caregivers were conducted without the presence of patients, were hold at the University center in 2017 and all were tape-recorded. We stopped data collection until saturation was reached.

#### Qualitative thematic analysis

Transcripts from the interviews were analyzed using a narrative approach [68–70] to identify - through a thematic analysis - thematic patterns in the data and to derive the preliminary conceptual framework of family caregiver engagement (namely, the Caregiving Health Engagement Model – CHE Model). Narrative inquiry is a well-established methodology particularly useful to collect human beings’ stories beyond literal description. Narrative researchers are interested in how the story is told in addition to its content. In-depth insights are gained by making sense of the finer differences between apparently similar stories.

The recorded interviews were transcribed verbatim and analyzed thematically, and key stories were identified. The narrative analytical process adopted in this research followed the hermeneutic tradition of continuously comparing the parts to the whole in a “hermeneutic circle”, allowing the researchers to interpret the text in depth and to identify the caregivers’ experience and feelings [71, 72]. Particularly, trhough thematic analysis of the collected narratives, two researchers (SB and GG) independently read through each interview multiple times to familiarize with the generated contents. Interviews were then independently reviewed and analyzed according to the principles of thematic analysis. Notably, the researchers were careful to make codes emerge from the data inductively. Following this process, the researchers allowed wider themes to emerge, with clear quotations of each theme taken from the data to illustrate and support the findings of the analysis. To confirm accuracy and interpretation of the data during the coding process and themes development, findings were discussed and agreed between two researchers (SB and GG) and also among the study steering group (SS, GG, CC, AB).

### Stage 2. Generation and psychometric evaluation of the caregiving Health engagement scale (CHE-s)

Building on the information collected in stage one and on the conceptual model derived from that information, in this second stage we operationalized them in a set of specific items. This stage included generating, refining, and preliminary testing the Caregiving Health Engagement Scale (CHE-s)‘s psychometric properties, inspired by the principles of the COSMIN guidelines [73]. The items of the scale were generated through formative qualitative research (stage 1), and a preliminary review of relevant literature. In this second stage, a convenience sample of family caregivers was used to assess the performance of the measure and to assess its validity. All statistics were performed using the statistical package IBM SPSS Statistics (Version 22.0; IBM Inc., New York, USA) [74]. A *p*-value of < 0.05 (two-tailed) was considered significant in all analyses.

#### Item generation, content and face validity assessment

We generated an item pool - originally formulated in Italian- to measure the construct of caregiver engagement using literature reviews or our own raw interview data from first stage of this study, that consisted of four dimensions rooted in the 4 profiles described by the caregiver engagement conceptualization (CHE model) emerged from the first qualitative stage (see results section for more details): “denial”; “hyper-activation”; and “drowning” and “balance” to be tested with 7-point scaling (see Table 2 for an overview of the items). The item pool was reviewed for content and face validity - according to the COSMIN guidelines - by the project steering committee, and by including some caregivers from the first stage, to check the content validity including the relevance, comprehensiveness and comprehensibility of the items, response options, and instructions and the final 7 items were refined. The CHE-s adopted an ordinal structure in order to be consistent with the CHE model’s conceptualization (developed in stage 1), which envisages four different profiles along the engagement continuum (see Results section). Although the CHE model described four engagement profiles, the ordinal scale was measured on a 7-point scale in order to facilitate caregivers’ responses and to avoid social desirability bias [75]. This scaling choice has been tested with success in previous studies on a patient engagement measure developed by the Authors of this work [76].

#### Sampling and recruitment procedures

A convenience sample of family caregiver was involved for the validation stage of this study. Eligibility criteria for family caregivers inclusion in this stage were the same of stage one: to 1) have been assisted a patient with activities of daily living and complex health care needs for at list 6 months; 2) aged over 18; 3) being a caregiver without cognitive impairment; 4) and able to speak and understand Italian. We excluded potential participants if they were unable to read and understand the Italian language. According to Tabachnick & Fidell [77], we established a minimum number of 70 participants as well as for principal components / exploratory factor analysis, a ratio of 10 participants per item is sufficient.

#### Data collection

Data collection occurred in 2018 through the administration of a structured questionnaire consisting of the items of the CHE-s and concurrent measures (see the next paragraph for further details) in addition to items on socio-demographics. The participants provided information on age, gender, relationship with the patients, the patients’ disease, years since the patients’ diagnosis. The questionnaire was designed as a self-administered questionnaire, according to standard protocols for questionnaire design and testing.

#### Concurrent measures

Due to the psycho-social nature of the new scale, the measure used for the validation process included the Caregiver Burden Inventory [55, 78] and the Revised Scale for Caregiver Self-Efficacy [59]. Following a detailed description of those instruments.

- *Caregiver Burden Inventory (CBI).* It is a multidimensional scale proposed to evaluate the impact of burden on different aspects of a caregiver’s life, reflecting various areas of the caregiver’s well-being and function which may be differentially affected by the relative’s medical condition [55]. Although the original scale proposes five dimensions, we identified four groups or factors, as suggested by Marvardi and colleagues [79]. The four burden dimensions are: time-dependent burden (T/dep-B, α = 0.92), evaluating stress caused by restriction of one’s personal time; psychophysical burden (Psy-Phys-B, α = 0.95), referring to a sense of failure regarding one’s hopes and physical stress; emotional burden (Emot-B, α = 0.74), concerning to any embarrassment or feeling of shame caused by the patient; and social burden (Soc-B, α = 0.94), caused by conflicts of roles concerning one’s job or family. In the present study, an Italian translation was adopted.
- *Revised Scale for Caregiving Self-Efficacy (SE)*. Self-efficacy has been conceptualized as a person’s belief about her or his ability to organize and execute courses of action to manage given situations [80]. When applied to the experiences of caregivers, it can explain family member’s ability to cope with chronic demands and challenges of caregiving. Developed by Steffen and colleagues [59], the 15-item Revised Scale for Caregiving Self-Efficacy (SE) is an assessment tool in clinical and research settings. It consists of three subscales which measures three domains of caregiving self-efficacy: SE-Obtaining respite (α = 0.93), which comprises behaviors that caregivers would initiate or participate in order to reduce their own distress and enhance well-being; SE-Controlling upsetting thoughts (α = 0.94), which is the ability to confront and regulate distressing and unhelpful thoughts about caregiving; SE-Responding to Disruptive Patient Behaviors (α = 0.95), which is the perceived capability to manage difficult patient behaviors. In the present study, an Italian translation was adopted.

### Statistical analysis

The number and frequency for categorical variables, and the mean and standard deviation for the continuous variables, were calculated as descriptive statistics.

Since the 7 proposed items had an ordinal nature, the data analysis involved suitable technique for ordinal data. In particular, the exploration of the factorial structure was carried out using a Categorical Principal Component Analysis (CATPCA) and its reliability was assessed using the Ordinal Alpha via Empirical Copula Index [81]. To test and verify the unidimensionality of the scale, (a) an exploratory CATPCA, (b) a confirmatory CFA for ordinal data, and (c) a Rasch Model were performed. Descriptive statistics of the individual items were calculated to conduct the initial exploration of the data.

## Results

Overall, 230 (32 caregiver involved in stage 1; 198 caregivers - not involving the ones of the first stage - involved in stage 2) caregivers participated in the study. Socio-demographic and clinical characteristics are summarized in Table 1.

**Table 1:** Demographic and clinical characteristics of the sample

Demographic variables (caregivers)		Stage 1 (*N* = 32) % (*N*)		Stage 2 (*N* = 198) % (*N*)
Mean age (s.d.)		62.9 (6.2)		53.3 (12.5)
Gender (% females)		58 (19)		76.5 (151)
Relationship with the patient (%)				
Parent		3.1 (1)		56.1 (111)
Son/daughter		12.5 (4)		13.6 (27)
Spouse/partner		84.4 (27)		24.7 (49)
Other		–		5.6 (11)
Clinical variables (patient)				
Mean years from diagnosis (s.d.)		4.9 (5.3)		11.4 (8.7)
Patient’s main disease (%)				
Pulmonary disease		21.9 (7)		38.4 (76)
Alzheimer’s disease		3.1 (1)		9.1 (18)
Diabetes		34.4 (11)		7.1 (14)
Cystic fibrosis		–		7.1 (14)
Cardiovascular disease		28.0 (9)		4.5 (9)
Rheumatoid arthritis		–		5.1 (10)
Cancer		6.3 (2)		4.5 (9)
Dementia		6.3 (2)		3.5 (7)
Connective tissue disease		–		2.5 (5)
Multiple sclerosis		–		1.5 (3)
Vasculitis		–		1.5 (3)
Other (various)		–		15.2 (30)

### Stage 1. Conceptualizing family caregiver engagement in healthcare: the Caregiving Health Engagement Model

Basing on the results from the caregivers’ interviews, we developed a conceptual definition of the psycho-social experience of family caregiver engagement in the healthcare process. In total, 32 family caregivers – of different ages and degree of relationship with the patient - were involved.

According to the collected narratives and to the thematic analysis of them, the family caregiver psycho-social experience of engagement has a processual and dynamical nature. Particularly, the thematic analysis revealed that this process features four main experiential profiles describing different ways of being engaged in the healthcare process. Such experiential profiles are not necessarily to be considered as a linear continuum: although each of them corresponds to a different level of family caregiver engagement, the psychological process to pass from one position to another can change in its trajectory- Thus, those different positions may be considered as alternative experiences of caregivers along the health journey of their loved one. Caregivers trajectories along the four position may change due to individual or contextual characteristics: some caregivers may proceed linearly along the continuum, someone may skip one or more positions, someone can go back, and someone may be stuck in the same position without evolving. The psycho-social experience of caregiving is complex and differs among individuals. The sequence of such experiential positions together with the length of them may vary across individuals. However, the model depicts a few common characteristics and experiences that may recur and help in better understanding the psychological experience of family carers when engaging in the care of an ill relative.

The following paragraphs show a detailed description of the different psycho-social positions of family caregiver engagement theorized in the CHE model. Each position will be substantiated by enucleating prototypical extracts from the family caregiver interviews. Four consistent types of narrative/storylines in the process of family caregiver engagement were identified: 1) *denial*, 2) *hyper-activation*, 3) *drowning* and 4) *balance*. The name of the four positions – describing the four main profiles of the CHE Model - comes from in-vivo codes from the narratives collected. Following an in-depth description of the four main profiles emerging from the thematic analysis, supported by extracts from the interviews with family caregivers.

#### Position 1: denial

This is the first position in the continuum of *family caregiving engagement,* often occurring at the beginning of the care pathway*,* such as at the moment of the diagnosis or in occasion of a worsening condition of the beloved one. Generally*,* family caregivers in this position experience a situation of emotional *shock* due to a sense of disorientation, the anxiety and the grief for the changed health condition of their ill relative.

*“I was not able to manage the shock” (wife, 47 years old)*

*“I can’t believe what happened…I still struggle in accepting that”(husband, 62 years old)*

For this reason, the caregiver tends to enact defensive psychological mechanisms such as avoidance (of thoughts related to the health condition of the patient and to the caregiver responsibilities), and anger. When in this position, caregivers often feel ineffective in their caregiving function thus resulting in the denial of that role.

*“Sometimes, I just want to run away”(son, 35 years old)*

*“This is not my duty. This is not my role”(wife, 54 years old)*

Caregivers in this position often feel unable to actively play the caregiving function and to assume their loved one’s care responsibility. Moreover, they expect to delegate the therapeutic decisions about their loved one to the clinical staff. Pragmatic support is transferred to other family member or to professional caregivers or in-home nurses. This not only means a delegation of pragmatic aspects related to caring but also a psychological avoidance of the caregiving role assumption. The main need of caregivers in this position is to be emotionally supported in order to elaborate the new role identity and to be helped in acquiring pragmatic and organizational skills to effectively manage the caregiving tasks.

#### Position 2: hyper-activation

This position is more typical of family caregivers who have started to accept the pragmatic and emotional changes resulting from the new health condition of their loved ones. Caregiver at this stage - unless they are acquiring an initial understanding of the health condition of their loved one and start to emotionally adapt to this new condition – often reports a feeling of psychological “alarm” and hyper-activation. However, as they have initial literacy and knowledge about the patient health condition, they take more care - compared to the previous stage – of their loved ones’ symptoms and are able to be proactive in managing it. The caregiving function is perceived as an emotional burden and makes caregivers worried and alarmed.

*“I found it very frightening at the start, very frightening. I couldn’t sleep”. (wife, 61 years old)*

*“Whenever I’ve been out and I am not there for him (the husband) the first thing I do is to look on the telephone, see if there’s any messages. I am always on alert”. (wife, 66 years old)*

Practical assistance offered by family caregivers experiencing this stage is often effective but, sometimes even excessive, because the caregiver tends to replace his/her loved one even in the tasks and actions that the latter would be able to do for himself/herself. In terms of emotional and psychological awareness, however, the caregiver is still in difficulty: he/she tends to deny or avoid the emotional burden due to empathy with the psychological difficulties of his/her relative; on the contrary, he/she prefers to shift the assistance and the relationship with his beloved on a practical level and denies or delegates to others the listening to the most emotional or psychological needs of his loved one, because it constitutes a major source of suffering.

*“I'm a dressmaker, I'm not a doctor. I do not know how to help my husband and I rely on doctors”(wife, 70 years old)*

As anticipated, the caring role is in this position responsibly assumed, but carried out in an overactive mode. The function of caregiving becomes all-encompassing and “draining” the physical and mental energies of the person.

#### Position 3. Drowning

In this position the family caregiver, thanks to the passage of time or the effective support received from the health system, has often matured a functional adaptation to the clinical condition of his beloved one. Although still tired on the emotional and psychological level, the caregiver in this position has been as able to develop adaptive strategies for an efficient management of caregiving activities. He/she is more organized, more able to read the care needs of his/her loved one and to respond to them effectively. The family caregiver feels even more legitimate to actively participate in the medical consultation and to play a more active role in the negotiation of therapeutic choices. However, he/she does not feel completely effective in coping with the caring needs of his/her loved one and brings difficulties and disorientation in the case of unexpected changes in the context of daily life (e.g.: in the case of travel, transfers, holiday periods, absence of carers, etc.) or the occurrence of events in the social context that challenge established strategies and assistance routines (eg: influence of the caregiver, etc.).

*“Taking care of my mum is a daily routine. This is currently my job and I feel ok with that. However I experience some troubles when I have to take some day off with my family or I have to go abroad for work. These episodes are really difficult to be managed”. (son, 37 years old)*

The caregiver in this position, in fact, despite having a good level of skills for the management of assistance needs of their loved one, feel insecure and with low levels of perceived self-efficacy. Moreover, he/she lives his own role of caregiving in an all-encompassing way, incapable of integrating it in a balanced way with his own life and self-expression needs.

*“Since my wife has become ill, my life is no longer life” (Husband, 64 years old)*

*“I lost my freedom” (Parent, 39 years old)*

*“You feel conditioned for life” (Son, 38 years old)*

*“I was no longer his wife. I was his nurse” (Wife, 64 years old)*

*“I am his point of reference. only if I am with him, he feels calm” (Wife, 71 years old)*

*“I gave up everything. I do not know who I am anymore (Son, 56 years old)*

#### Position 4. Balance

Fully engaged caregivers have often matured a more positive vision of their role. They generally became more and more aware of the need to get on with their own life. The caregiver experiencing this position not only shows full autonomy in responding to the caring needs of his loved one, but he also feels more capable and effective and is more confident of his skills. He/she also managed to consolidate a good partnership relationship with the care team with whom he/she relate with greater proactivity and collaboration. In terms of identity, the caregiver in this position managed to find a higher balance and a more integrated vision of the different tasks of life and the different roles to which he/she is called to perform. The caregiving function is therefore lived in a less totalizing way because, although dedicating himself/herself to this activity with commitment, he/she becomes able to allow himself/herself spaces dedicated to self-care. The maintenance, for example, of hobbies, good interpersonal relationships, work duties or roles in the community of reference are all indicators of the ability of the caregiver not only to effectively fulfill the needs of assistance of his/her beloved one, but also to know how to maintain space and pleasure in personal activities that feed motivation and energy (including the psychic one) ​​necessary to proceed in the healthcare journey of the patient.

*“I can’t let myself go in any way because it wouldn’t be right”. (Husband, 70 years old)*

In this case, they are generally more knowledgeable caregivers and more able to understand the information concerning the clinical condition and the medical prescriptions of their loved one. They are more open and available caregivers to participate in education and awareness initiatives offered by the referring healthcare system. Finally, when in this position, family caregivers are also able to become the driving force of other people who live similar care experiences, motivating them and suggesting good strategies for managing and adapting to the caring role

*“My husband did everything at home. I had to change skin and reinvent my life” (wife, 72 years old)*

*“We slowly returned to being a husband and wife” (wife, 76 years old)*

### Stage 2. Generation and psychometric evaluation of the caregiver Health engagement scale (CHE-s)

Based on the caregiving health engagement developed in stage 1, a list of statements has been developed for the CHE-s. For face and content validation, the questionnaire was pretested among 12 caregivers from the first stage. According to their responses the items were clear and easy to understand, so that no changes have been made. Each item included in the final version of the scale, as described in Table 2, allows caregivers to position themselves in the state that mostly describes their current experience. Each choice theoretically corresponds to one of the engagement position described in the CHE model (see previous paragraphs). Lower scores corresponds to the first and second positions of the CHE-Model; higher scores to the third and fourth ones. More details about the scoring system are available upon request to the authors.

**Table 2:** CHE-s items’ overview

	“Denial”	“Hyper-activation”	“Drowning”	“Balance”
CHE_1	I feel in blackout	I feel alert	I am aware	I’m positive
CHE_2	I feel lost	I’m in alarm	I am conscious	I feel serene
CHE_3	I feel overwhelmed by emotions	I’m anxious every time a new symptom appears	I feel to have adjusted to my loved one’s illness	I have a sense of consistency and continuity in my life despite his/her illness
CHE_4	I feel totally crushed by the disease	I distress a lot when a new symptom appears	Overall I feel I have accepted his/her illness	I can make sense of my life despite his/her illness
CHE_5	I cannot understand what my loved one would need to get better	I understand what my loved one would need, but I cannot help him/her	I understand what my loved one would need and sometimes I can help him/her	I can anticipate the needs of my loved one and help him/her effectively
CHE_6	Right now I do not feel able to assist my loved one alone	Assisting my loved one absorbs my time and energy	I found an acceptable balance between the need for assistance of my loved-one and my daily activities	I feel able to manage my life projects and the assistance to my loved one in a renewed normality
CHE_7	I delegate to the health professionals the decisions related to the care of my loved one	I need constant confirmations by the health providers to make decisions about my loved one’s care	I can recognize when it is necessary to contact the health professionals to make a decision about his/her treatment	I actively collaborate with my loved one’s health professionals

Table 3 shows descriptive statistics of all items, as well as their Shannon Entropy index. Table 4 provides the inter-item polychoric correlation matrix (Pearson, 1900), which is a measure of bivariate association arising when observed variables are ordered categorical variables derived from polychotomizing latent underlying continuous variables. The average inter-item polychoric correlation is a subtype of internal consistency reliability. It is obtained by taking all of the items on a test that probes the same construct, determining the polychoric correlation coefficient for each pair of items and finally taking the average of all of these polychoric correlation coefficients. Every polychoric correlation coefficient was higher than 0.52. The average inter-item polychoric correlation is equal to 0.66, which indicates a high correlation between items.

**Table 3:** Item-level descriptive statistics for ranks on the CHE 7-item scale

CHE-s item	Rank range	Min	Max	Median	Shannon entropy
CHE_1	1–4	1	4	3	0.93
CHE_2	1–4	1	4	3	0.90
CHE_3	1–4	1	4	3	0.98
CHE_4	1–4	1	4	4	0.94
CHE_5	1–4	2	4	4	0.87
CHE_6	1–4	2	4	4	0.94
CHE_7	1–4	1	4	3	0.90

**Table 4:** Item-item polychoric correlation matrix for ranks on the CHE

	CHE_1	CHE_2	CHE_3	CHE_4	CHE_5	CHE_6	CHE_7
CHE_1	–	0.86	0.75	0.67	0.68	0.65	0.54
CHE_2		–	0.78	0.72	0.66	0.65	0.59
CHE_3			–	0.78	0.66	0.7	0.52
CHE_4				–	0.63	0.68	0.58
CHE_5					–	0.64	0.62
CHE_6						–	0.55
CHE_7							–

#### Exploratory categorical principal component analysis

An exploratory categorical principal component analysis (CATPCA) was conducted. An initial analysis was performed without any restriction on the number of metric factors to be estimated. The initial analysis yielded one factor with eigenvalue of 4.69, which is over the Kaiser Criterion of 1, explaining 67.0% of the total variability. The CATPCA results, together with the ordinal alpha of 0.88, suggests a monodimensional latent structure and a very good internal consistency. Table 5 shows the factor loadings for the one solution.

**Table 5:** Factor loadings from CATPCA – one factor solution. All factor loadings had a very high value (> 0.7), confirming the unidimensionality of the scale

CHE-s item	One factor solution
CHE_1	0.86
CHE_2	0.86
CHE_3	0.86
CHE_4	0.85
CHE_5	0.79
CHE_6	0.79
CHE_7	0.70

#### Confirmatory factorial analysis

**Fig. 2:**
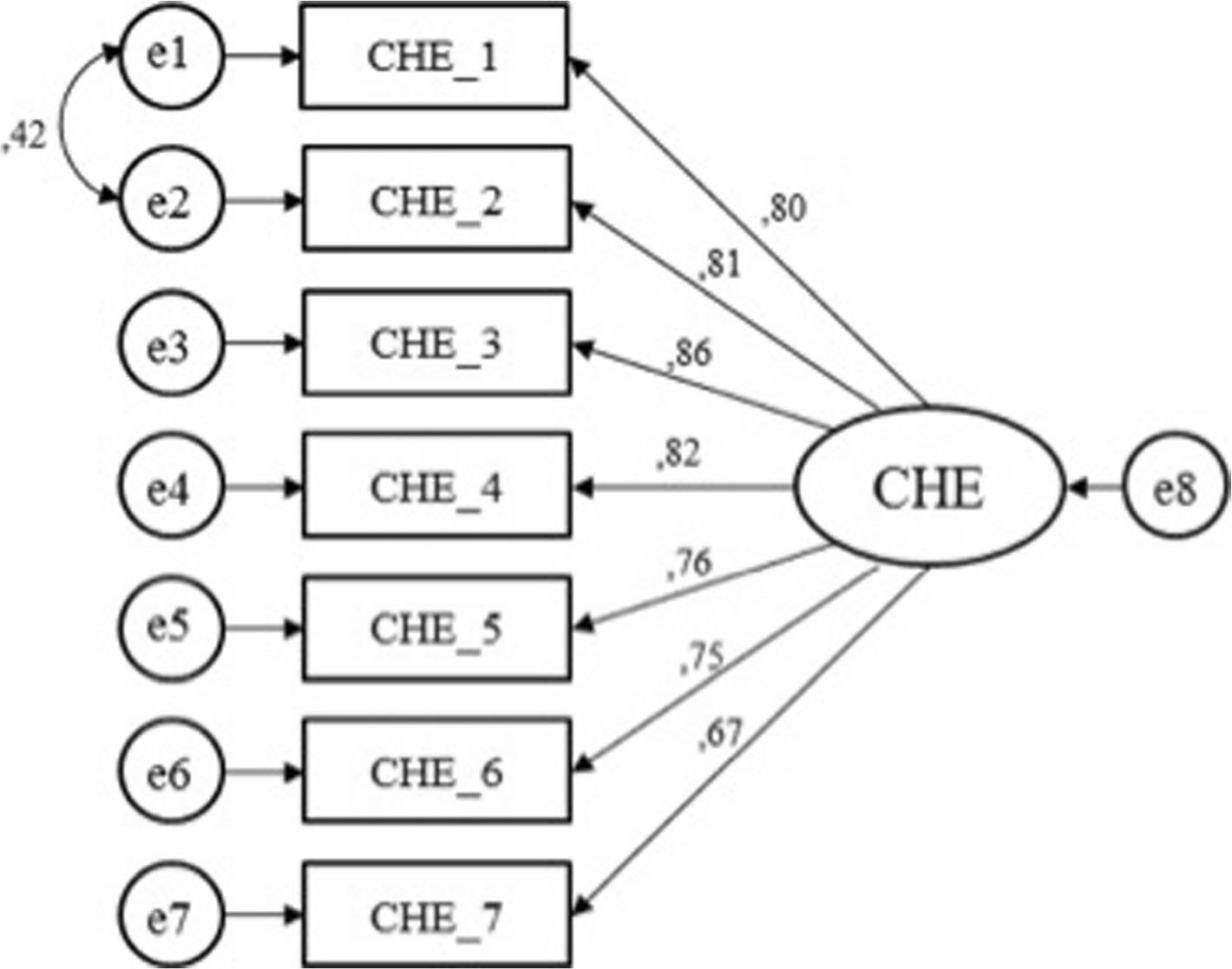
CFA on CHE-s: Standardized estimates

A Confirmatory Factor Analysis (CFA) was performed (Fig. 2). The estimation method was asymptotically distribution free, particularly suitable for ordinal data non not-Gaussian distributions. To evaluate the closeness of the hypothetical model to the empirical data, multiple goodness-of-fit indexes were used, including the ratio of the Chi-square to degrees of freedom (χ2/df), the Comparative Fit Index (CFI), the Standardized Root Mean Square Residual (SRMR), and the Root Mean Square Error of Approximation (RMSEA). To test the model, each variable was allowed to load on only one factor, and one variable loading in the latent factor was fixed at 1.0. For the remaining factor loadings, residual variances were freely estimated.

CFA showed adequate goodness of fit indices. The fit indices met the criteria of fit for the hypothesized one-factor structure. Chi square value (χ2 = 18.81, df = 13, p = n.s.) and goodness of fit indices (CFI = 0.96, RMR = 0.03, RMSEA = 0.05) suggested that the model is coherent with the data.

#### Rasch model

A Rasch Model was implemented to further investigate whether the CHE-s was uni-dimensional, and whether all items fit the model well.

Two (infit and outfit) mean square (MNSQ) statistics were computed to check whether the items fit the expected model. MNSQ determines how well each item contributes to defining a single underlying construct (uni-dimensionality). If the data fitted the Rasch Model, the fit statistics should be between 0.6 and 1.4 (in clinical statistics the fit could be between 0.5 and 1.5, Wright et al., 1994). Table 6 shows the results of the Rasch Analysis. The measure of each item represents the estimate for the item difficulty expressed in logits; SEM is the standard error of measurement in estimation of the item difficulty; Infit and Outfit are measurement of item fit.

**Table 6:** CHE-s – Rasch Analysis

CHE item	Measure (logits)	SE	Infit MNSQ	Outfit MNSQ
CHE_1	1.213	0.3	0.751	0.775
CHE_2	1.076	0.3	0.671	0.663
CHE_3	1.077	0.3	0.736	0.768
CHE_4	0.886	0.3	0.815	0.813
CHE_5	0.330	0.4	0.930	0.963
CHE_6	0.574	0.4	1.004	0.968
CHE_7	0.409	0.3	1.274	1.240

Infit and Outfit statistics ranged from 0.66 to 1.27, which all are within the acceptable range. The person separation index (PSI) was calculated to evaluate the reliability in the Rasch Model (PSI = 0.907). Rasch Model confirmed the unidimensionality of CHE-s and the fit of each item of the scale to the data.

#### Concurrent validity

To assess concurrent validity, CHE-s factor scores were evaluated in relation to CBI (Caregiver Burden Inventory) and SE (Caregiving Self-Efficacy) scores.

First, a Pearson correlation was calculated between CHE-s and CBI factors. The results showed a moderate correlation between Caregiver Engagement and all four burden dimensions: T/dep-B (r = − 0.40, *p* < 0.001); Psy-Phys-B (r = − 0.62, *p* < 0.001); Emot-B (r = − 0.43, *p* < 0.001); and Soc-B (r = − 0.42, *p* < 0.001). In other words, higher levels of caregiver engagement are significantly related to lower levels of caregiving burden, in terms of both restriction of one’s personal time, psycho-physical stress, emotional and social concerns. This result is in accordance with theoretical expectations.

Next, a Pearson correlation was calculated between CHE-s and SE factors. The results showed a moderate correlation between Caregiver Engagement and two dimensions of caregiving self-efficacy: SE-Obtaining respite (r = 0.25, *p* < 0.001) and SE-Responding to Disruptive Patient Behaviors (r = 0.48, *p* < 0.001). This means that higher levels of caregiver engagement are significantly related to a higher ability to reduce one own distress and enhance wellbeing, as well as a higher capability to manage difficult patient behaviors. No significant relation was found with the third dimension of caregiving self-efficacy, SE-Controlling upsetting thoughts, which is the ability to confront and regulate distressing thoughts about caregiving.

## Discussion

This study describes the development of a new, brief, self-report assessment tool for measuring family caregiver engagement in healthcare called the CHE-s and includes an evaluation of the psychometric properties of the CHE-s among a convenience sample of family caregivers.

The first stage of this study was exploratory and was aimed to conduct a qualitative inquiry – according to a narrative approach - to identify the inner nature and the factors involved in the experience of family caregiver engagement in healthcare. The second stage was devoted to the development and first validation of a psychometric tool in order to measure the dynamic experience of caregiver engagement in healthcare, rooted in the conceptual model developed in stage 1.

The first qualitative stage of the study constituted the basis for the conceptual framework of the CHE-s instrument. This phase of the study confirmed that family caregivers often play the role of crucial “mediator” in the relationship between the patient and the healthcare team, as showed by other authors [82]. Moreover, the study showed that family caregivers that are engaged in the healthcare process are the ones succeeding in *adapting* to the patients’ illness and in *reframing* their role in reaching balance between their caring role and their life. This evidence is coherent with the literature on “adaptation theories” which explains the psychological process of adjustment that people undergo whenever facing life events that require them to undertake a major revision of their assumptions about the world and to adapt to those situations (eg, the onset of a disease of a relative). One of the most established among these models is the one developed by Kubler-Ross [83, 84] who proposed a five-stage theory describing the psychological reactions of terminally ill individuals to their disease: that is, denial–dissociation–isolation, anger, bargaining, depression, and acceptance. This theory, although focused on the patients, can be surely linked with our results on family caregivers adjustment processes. Moreover, according to our results, engaged caregivers act as actual partners of the healthcare providers, as described also in other recent studies [85, 86].

Furthermore, the experience of caregiver engagement is not an on/off status, as if it could be activated simply with a simple and circumscribed intervention. On the contrary, caregiver engagement emerged to be a psychosocial process resulting from a dynamic path of maturation and reframing of the individual caregiver’s meaning-making about his/her role along the patients’ healthcare pathway. As for the patient, even for the family caregiver the change in the health conditions of one’s loved one has a relevant emotional impact [87, 88]. In our study, family caregivers often reported not only a strong emotional weight due to the diagnosis of their loved one, but also to the effort of enduring in the rest of their daily routines and relationships. In the clinical-psychological literature, this (critical) dimension of the caring experience is usually labeled in terms of “burden”, and the negative and draining impact of these experiences on caregivers’ psychological and physical energy is widely discussed [6, 34, 48, 89–92]. However, according to our study, this emotional burden is only one of the psychological components of the caregivers’ engagement experience. Another crucial dimension emerged from our study is the ability to adequately read and empathize with the care needs of the ill relative [14, 93, 94]. This aspect is described by caregivers as a skill that improves over time and that requires a basic knowledge about the clinical conditions of their loved ones. This appears aligned with previous researches, which demonstrated how this caregivers’ mirroring function depends on their psychological resources and energies [95–97]. Furthermore, previous research has demonstrated how learning to adequately respond to the healthcare and social needs of one own loved one is a long psychological process, which needs to be supported and guided by health workers [1, 98, 99].

Moreover, the interviewed caregivers described their engagement in caregiving tasks as dependent on their ability to find a psychological balance. This aspect is also discussed in other studies as crucial in guaranteeing caregivers the possibility to effectively adjust to their caring role [100–102]. Often the caregiver has to find a new balance between the care needs of their loved ones and their personal and private needs and priorities. Moreover, the person who assumes the role of caregiver along a patient journey is often the same person who assists other family members, such as children or other needy figs [103].. Clinical and psychological literature have largely demonstrated how much important it is to support caregivers in achieving an appropriate balance between the ability to reflect the caring needs of their loved one and the ability to listening and responding to their own health and life needs [1, 97].

Finally, similarly to what happens for patient engagement dynamics, also in the case of the family caregiver the engagement journey requires a process of self-identity reconfiguration [76, 104], in direction of a more proactive and participatory attitude towards the relationship with the healthcare system [11]. In other words, in the engagement process also the caregiver is called to opt for a role of major centrality in the path of care: this involves understanding what are the areas of action, the requirements and the skills inherent to the caregiving role, but also assuming such role in a clear way and sharing it with both the health providers and other members of the family/peer group [105]. This is not automatic, but depends on a complex psychological process of elaboration and synergic integration of this “new role of caregiver” with the other “roles” included in the individual’s self-identity. For instance, the role of worker, that of partner/relative and any other roles played by the person in his/her reference context [53, 106].

**Fig. 3:**
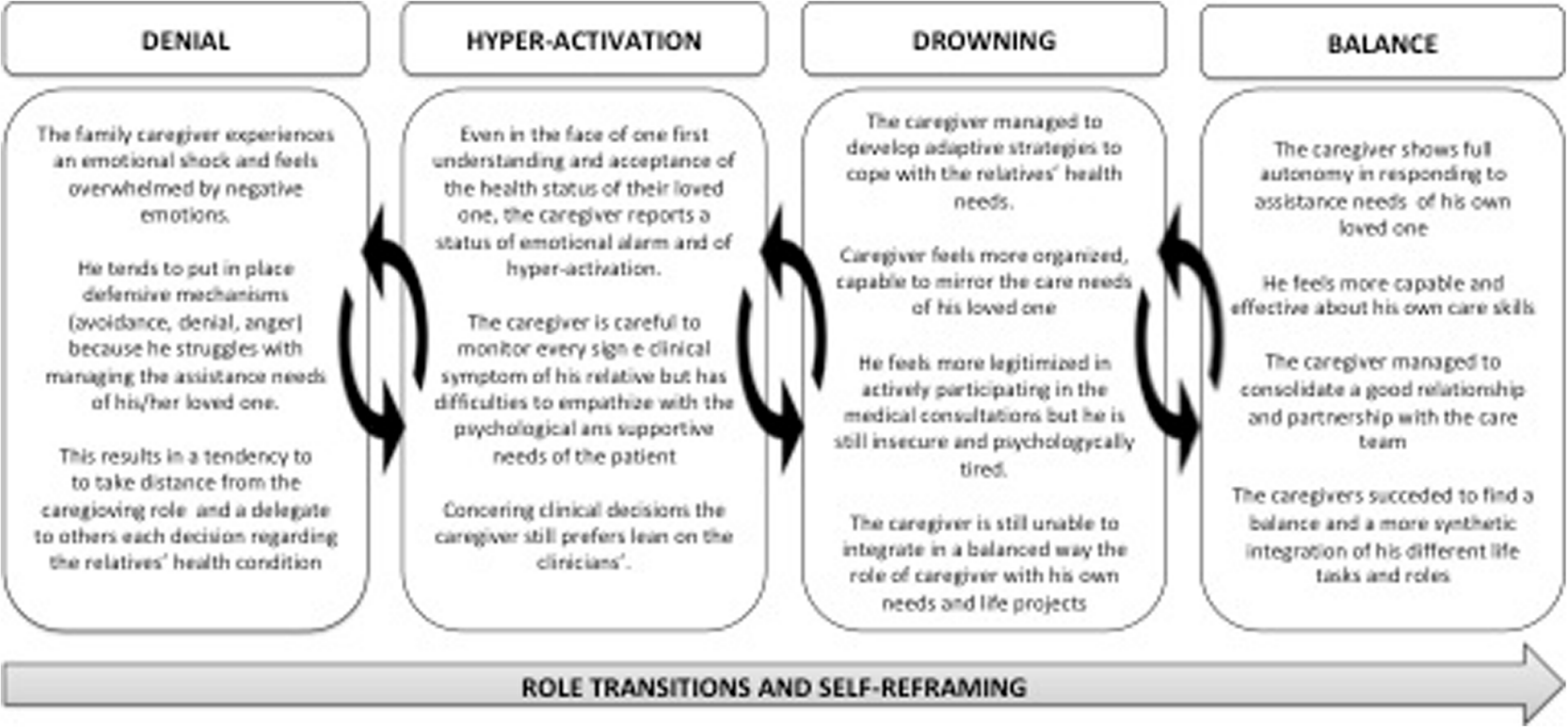
The process of Family Caregiver Engagement as described in the CHE model

The interlacement of these subjective dimensions emerged from the in-depth study of the family caregiver experience of engagement results into four distinctive types of storylines in the process of family caregiver engagement (see Fig. 3), namely: 1) *denial:* caregivers in this position tend to deny and reject their role of caregiving, 2) *hyper-activation*: caregivers in this position are constantly on alert and find difficult to effectively recognize the needs of their loved one; 3) *drowning:* caregivers in this position have developed effective care strategies but struggle to find a balance between their caregiver role and their life needs and projects; and 4) *balance:* caregivers in this position perceived themselves as effective and confident in their role of care and have gained greater balance and better integration of their different life roles and tasks.

The processual and multifaceted experience of family caregiver engagement in healthcare has been theorized in the CHE model (see Results section) as schematized in Fig. 3.

Due to the complex and evolutionary nature of the caregiver engagement process described by the CHE theory, the development of a psychometrically validated tool to assess this caregivers’ experience was needed. The CHE-s, indeed, translates the insights collected in the stage 1 of this study and embodies the dimensions, which emerged to be fundamentals of the family caregiver’s engagement experience.

Overall, the results provide solid evidence for the internal consistency and concurrent validity of the CHE-s. Unique features of this measure are that the items were derived from a theoretical model developed according to the direct caregivers’ experience of engagement in healthcare, the response format is ordinal on a 7-point scale, and the CHE-s includes items that are designed to help clinicians and researchers to identify specific psycho-social needs that may be targeted for delivering more personalized supportive actions. The CHE-s is a simple, valid, easily interpreted, and reproducible tool for assessing family caregiver engagement in healthcare across disease conditions and monitoring aspects of the caregiving function that are important to family caregivers. The measure captures the fluctuating and dynamic nature of caregiver engagement, resulting in a punctual family caregiver’s profile of engagement.

The CHE-s is based on caregivers’ views and perspectives of what constitutes the experience of being engaged in the healthcare of a loved one rather than what researchers or health providers presume to be important for defining caregiver engagement. The qualitative base makes the CHE-s particularly relevant to person-centered services and approaches to care.

The findings suggest that the CHE-s is a relevant and comprehensible tool, as well as a valuable addition for clinical practice to evaluate family caregiver engagement in healthcare.

Due to its cross-disease nature, this new measure provides a potentially useful tool for epidemiological studies, a guide for the allocation of health care service resources in responding to the caregiver needs and an improvement of our understanding of the factors contributing to caregiver engagement. This might also allow clinicians to develop and provide educational intervention for caregiver engagement which are valid and applicable across clinical settings.

The current study has several limitations that should be noted. First, participants were recruited using a convenience sampling, which may have influenced the results obtained, since only those more interested may have participated. In future work it will be necessary to assess the generalization of the results, evaluating the CHE-s usefulness and psychometric properties in other caregivers’ samples and contexts. Moreover, results suggest some possible ceiling effects in the CHE-s scores. The ceiling effect could be related to the sample characteristics but this aspect requires further research. The convenience sample, moreover, prevents us from calculating a response rate as it is unknown how many eligible participants were invited to be involved in the stage 2.

## Conclusion

The present results support the preliminary validity of the CHE-s, and further research on the measure appears warranted. Although further research on the scale with other samples is needed, findings of the studies reported in this paper provide preliminary evidence for its reliability and validity, and highlight possibilities for its broader application.

In an era of increased demand and value for the engagement in the healthcare process, a reliable instrument that allows family caregivers across clinical settings to assess their ability to be actively engaged in their caring role can produce improvement of health services and medical education initiatives. Health care systems that prioritize person-led care may benefit from using the CHE-s to assess the level of engagement of family caregivers when performing their assistance role and designing interventions dedicated to support caregiver engagement [107].

## Data Availability

The datasets analysed during the current study are available from the corresponding author on reasonable request.

## Abbreviations

CHE Model: Caregiving Health Engagement Model
CHE-s: Caregiving Health Engagement Scale

## References

[1] Reinhard SC, Given B, Petlick NH, Bemis A. Supporting Family Caregivers in Providing Care. Patient Saf Qual An Evidence-Based Handb Nurses. 2008; 21328765

[2] Hughes N, Locock L, Ziebland S. Personal identity and the role of “carer” among relatives and friends of people with multiple sclerosis. Soc Sci Med. 2013. 10.1016/j.socscimed.2013.07.023 PMC3778435 24034954

[3] Bettio F, Verashchagina A, Camiileri- Cassar Alena Křížková Anne Lise Ellingsaeter Norway F, Sjørup Ania Plomien Marre Karu Virginia Ferreira Hanna Sutela Lucian-Liviu Albu Rachel Silvera Magdalena Piscová K, Bahna Friederike Maier M, Carl Aleksandra Kanjuo-Mrčela Maria Karamessini Elvira González Gago Maria Frey Anita Nyberg Sigurdur Johannesson Iceland A-H, et al. Long-Term Care for the elderly. Provisions and providers in 33 European countries EU Expert Group on Gender and Employment (EGGE). 2012.

[4] Bureau of Labor Statistics. Unpaid eldercare in the united states - 2013-14. United States Department of Labor. 2015.

[5] Qiu X, Sit JWH, Koo FK. The influence of Chinese culture on family caregivers of stroke survivors: a qualitative study. J Clin Nurs. 2018. 10.1111/jocn.13947 28677123

[6] National Center on Caregiving. Caregiver Health | Family Caregiver Alliance. Family Caregiver Alliance. 2015.

[7] Guerriere DN, Zagorski B, Coyte PC (2010). Care for the Family Caregiver : a place to start.

[8] Brémault-Phillips S, Parmar J, Johnson M, Huhn A, Mann A, Tian V, et al. The voices of family caregivers of seniors with chronic conditions: a window into their experience using a qualitative design. Springerplus. Springer International Publishing; 2016;5(1). 10.1186/s40064-016-2244-z PMC4870523 27330886

[9] Lévesque L, Ducharme F, Caron C, Hanson E, Magnusson L, Nolan J, et al. A partnership approach to service needs assessment with family caregivers of an aging relative living at home: a qualitative analysis of the experiences of caregivers and practitioners. Int J Nurs Stud. 2010. 10.1016/j.ijnurstu.2009.12.006 20189565

[10] Yeh H-Y, Ma W-F, Huang J-L, Hsueh K-C, Chiang L-C (2016). Evaluating the effectiveness of a family empowerment program on family function and pulmonary function of children with asthma: a randomized control trial. Int J Nurs stud [Internet]. Elsevier Ltd.

[11] Sadak T, Korpak A, Borson S. Measuring caregiver activation for health care: Validation of PBH-LCI:D. Geriatr Nurs [Internet]. Mosby Inc.; 2015 Jan [cited 2016 Jan 5];36(4):284–292. Available from: http://www.scopus.com/inward/record.url?eid=2-s2.0-84938739539&partnerID=tZOtx3y1 10.1016/j.gerinurse.2015.03.003 25959036

[12] White CL, Overbaugh KJ, Pickering CEZ, Piernik-Yoder B, James D, Patel DI, et al. Advancing Care for Family Caregivers of persons with dementia through caregiver and community partnerships. Res Involv Engagem. 2018; 10.1186/s40900-018-0084-4 PMC5776762 29387453

[13] Provenzi L, Barello S, Graffigna G. Caregiver engagement in the neonatal intensive care unit: Parental needs, engagement milestones, and action priorities for neonatal healthcare of preterm infants. In: Patient Engagement: A Consumer-Centered Model to Innovate Healthcare. 2016. p. 94–107.

[14] Barello S, Savarese M, Graffigna G. The role of caregivers in the elderly healthcare journey: insights for sustaining elderly patient engagement. Patient Engagement: A Consumer-Centered Model to Innovate Healthcare. 2016.

[15] Carole L. White KJO, Pickering CEZ, Piernik-Yoderz B, James D, Patel Darpan I. Puga F, Cleveland LF and J. Advancing Care for Family Caregivers of persons with dementia through caregiver and community partnerships. Res Involv Engagem. 2018 10.1186/s40900-018-0084-4; 10.1186/s40900-018-0084-4 PMC5776762 29387453

[16] Nucera A. An invisible and essential role for the community: The caregiver. Int J Stroke. 2016;

[17] Griffin JM, Friedemann-Sánchez G, Jensen AC, Taylor BC, Gravely A, Clothier B, et al. The invisible side of war: families caring for US service members with traumatic brain injuries and polytrauma. J Head Trauma Rehabil. 2012. 10.1097/HTR.0b013e3182274260 21873883

[18] Wolff JL, Roter DL. Hidden in plain sight: medical visit companions as a resource for vulnerable older adults. Arch Intern Med. 2008. 10.1001/archinte.168.13.1409 18625921

[19] Wolff JL, Spillman B. Older adults receiving assistance with physician visits and prescribed medications and their family caregivers: prevalence, characteristics, and hours of care. J Gerontol B Psychol Sci Soc Sci. 2014. 10.1093/geronb/gbu119 PMC4303066 25342825

[20] Almborg AH, Ulander K, Thulin A, Berg S. Discharge planning of stroke patients: the relatives’ perceptions of participation. J Clin Nurs. 2009. 10.1111/j.1365-2702.2008.02600.x 19239664

[21] Hickey M. What are the needs of families of critically ill patients? A review of the literature since 1976. Hear Lung 1990; 19(4):401-415 2196246

[22] Levine C, Halper D, Peist A, Gould DA (2010). Bridging troubled waters: family caregivers, transitions, and long-term care. Health Aff.

[23] Levine C, Feinberg L. Transitions in Care: are they person- and family-centered? Gener J am Soc aging. 2012;

[24] Kaye HS, Harrington C, Laplante MP (2010). Long-term care: who gets it, who provides it, who pays, and how much?. Health Aff.

[25] Newcomer RJ, Kang T, Doty P (2012). Allowing spouses to be paid personal care providers: spouse availability and effects on medicaid-funded service use and expenditures. Gerontologist..

[26] Hasselkus BR, Murray BJ (2007). Everyday occupation, well-being, and identity: the experience of caregivers in families with dementia. Am J Occup Ther.

[27] Rodday AM, Pedowitz EJ, Mayer DK, Ratichek SJ, Given CW, Parsons SK. Parental caregiving of children prior to hematopoietic stem cell transplant. Res Nurs Health [Internet]. 2012 Aug [cited 2016 Jan 5];35(4):328–39. Available from: http://www.scopus.com/inward/record.url?eid=2-s2.0-84863841717&partnerID=tZOtx3y1 10.1002/nur.21485 PMC4160236 22549793

[28] DeCamp LR, Leifheit K, Shah H, Valenzuela-Araujo D, Sloand E, Polk S (2016). Cross-cultural validation of the parent-patient activation measure in low income Spanish- and English-speaking parents. Patient Educ Couns.

[29] Provenzi L, Barello S, Graffigna G. Caregiver engagement in the neonatal intensive care unit: parental needs, engagement milestones, and action priorities for neonatal healthcare of preterm infants. Patient Engagement: A Consumer-Centered Model to Innovate Healthcare. 2016.

[30] Boehmer KR, Egginton JS, Branda ME, Kryworuchko J, Bodde A, Montori VM (2014). Missed opportunity? Caregiver participation in the clinical encounter. A videographic analysis. Patient Educ Couns.

[31] Beals KP, Wight RG, Aneshensel CS, Murphy D a, Miller-Martinez D (2006). The role of family caregivers in HIV medication adherence. AIDS Care.

[32] Rodriguez KM (2013). Intrinsic and extrinsic factors affecting Patient engagement in diabetes self-management: perspectives of a certified diabetes educator. Clin Ther.

[33] Dy SM, Purnell TS (2012). Key concepts relevant to quality of complex and shared decision-making in health care: a literature review. Soc Sci Med.

[34] Dobbins JF. Voicing care: discourse, identity and the making of family caregivers. Dissertation Abstracts International: Section B: The Sciences and Engineering 2009.

[35] Carman KL, Dardess P, Maurer M, Sofaer S, Adams K, Bechtel C (2013). Patient and family engagement: a framework for understanding the elements and developing interventions and policies. Health Aff (Millwood).

[36] Swartwout E, Drenkard K, McGuinn K, Grant S, El-Zein A. Patient and Family Engagement Summit. JONA J Nurs Adm [Internet]. 2016;46(Supplement):S11–S18. Available from: http://content.wkhealth.com/linkback/openurl?sid=WKPTLP:landingpage&an=00005110-201603001-00003 10.1097/NNA.0000000000000317 26906687

[37] Carpentier N. Caregiver identity as a useful concept for understanding the linkage between formal and informal Care systems: a case study. Sociol Mind. 2012.

[38] Bevan JL, Pecchioni LL. Understanding the impact of family caregiver cancer literacy on patient health outcomes. Patient Education and Counseling. 2008. 10.1016/j.pec.2008.02.022 18372142

[39] Wolff JL, Darer JD, Larsen KL (2016). Family Caregivers and consumer Health information technology. J Gen Intern Med.

[40] Bertoni A, Donato S, Graffigna G, Barello S, Parise M (2014). Engaged patients, engaged partnerships: singles and partners dealing with an acute cardiac event. Psychol Health Med [Internet]..

[41] Brown SM, Rozenblum R, Aboumatar HJ, Fagan M, Milic MM, Lee B (2015). Defining Patient and Family Engagement in the Intensive Care Unit. 358. Am J Respir Crit Care Med.

[42] Olding M, McMillan SE, Reeves S, Schmitt MH, Puntillo K, Kitto S. Patient and family involvement in adult critical and intensive care settings: a scoping review. Health Expect. 2016. 10.1111/hex.12402 PMC5139045 27878937

[43] Graffigna G, Barello S, Riva G, Castelnuovo G, Corbo M, Coppola L (2017). Recommandation for patient engagement promotion in care and cure for chronic conditions. Recenti Prog Med.

[44] Graffigna G, Barello S, Riva G, Savarese M, Menichetti J, Castelnuovo G, et al. Fertilizing a patient engagement ecosystem to innovate healthcare: Toward the first Italian Consensus conference on patient engagement. Front Psychol. 2017;8(JUN):1–6. 10.3389/fpsyg.2017.00812 PMC5460315 28634455

[45] Graffigna G, Barello S, Bonanomi A, Lozza E, Hibbard J (2015). Measuring patient activation in Italy: Translation, adaptation and validation of the Italian version of the patient activation measure 13 (PAM13-I). BMC Med Inf Decis Mak.

[46] Maurer M, Dardess P, Carman KL, Frazier K, Smeeding L. Guide to Patient and family engagement: environmental scan report. American Institutes for Research 2012.

[47] Graffigna G, Barello S, Libreri C, Bosio C a . How to engage type-2 diabetic patients in their own health management: implications for clinical practice. BMC public Health [Internet]. 2014;14:648. Available from: http://www.ncbi.nlm.nih.gov/pubmed/24966036. 10.1186/1471-2458-14-648 PMC4083034 24966036

[48] Barello S, Graffigna G, Savarese M, Bosio AC. Engaging patients in health management: Towards a preliminary theoretical conceptualization. Psicol della Salut [Internet]. Dipartimento di Architettura e Pianificazione; 2014;(2):11–33. Available from: http://www.scopus.com/inward/record.url?eid=2-s2.0-84919909427&partnerID=tZOtx3y1

[49] Barello S, Triberti S, Graffigna G, Libreri C, Serino S, Hibbard J, et al. eHealth for patient engagement: A Systematic Review. Vol. 6, Frontiers in Psychology. 2016. 10.3389/fpsyg.2015.02013 PMC4705444 26779108

[50] Menichetti J, Graffigna G. How older citizens engage in their health promotion: a qualitative research-driven taxonomy of experiences and meanings. BMJ Open. 2016. 10.1136/bmjopen-2015-010402 PMC4947780 27417196

[51] Blow FC, Oslin DW, Slaymaker SJ. Family and caregiver involvement in older adult addiction treatment initiation and engagement. Alcohol Clin Exp Res. 2017;

[52] Burke RE, Johnson-Koenke R, Nowels C, Silveira MJ, Jones J, Bekelman DB (2016). Can we engage caregiver spouses of patients with heart failure with a low-intensity, symptom-guided intervention?. Hear lung J acute Crit Care.

[53] Burke RE, Jones J, Ho PM, Bekelman DB. Caregivers’ perceived roles in caring for patients with heart failure: what do clinicians need to know? J Card Fail [Internet]. 2014 Oct [cited 2014 Oct 15];20(10):731–8. Available from: http://www.sciencedirect.com/science/article/pii/S1071916414006848 10.1016/j.cardfail.2014.07.011 25084216

[54] Losada A, Márquez-González M, Peñacoba C, Romero-Moreno R. Development and validation of the caregiver guilt questionnaire. Int Psychogeriatrics. 2010; 10.1017/S1041610210000074 20170587

[55] Novak M (1989). Application of a multidimensional caregiver burden inventory. Gerontologist..

[56] Eifert EK. Measuring caregiver identity: scale development and validation. ProQuest Dissertations and Theses 2014.

[57] Barbic SP, Bartlett SJ, Mayo NE. Emotional vitality in caregivers: application of Rasch Measurement Theory with secondary data to development and test a new measure. Clin Rehabil [Internet]. SAGE Publications Ltd; 2015 Jul [cited 2016 Jan 5];29(7):705–16. Available from: http://www.scopus.com/inward/record.url?eid=2-s2.0-84937033330&partnerID=tZOtx3y1 10.1177/0269215514552503 25246610

[58] Degeneffe CE, Chan F, Dunlap L, Man D, Sung C (2011). Development and validation of the caregiver empowerment scale: a resource for working with family Caregivers of persons with traumatic brain injury. Rehabil Psychol.

[59] Steffen AM, McKibbin C, Zeiss AM, Gallagher-Thompson D, Bandura A (2002). The revised scale for caregiving self-efficacy: reliability and validity studies. J Gerontol Ser B Psychol Sci Soc Sci.

[60] Deeken JF, Taylor KL, Mangan P, Yabroff KR, Ingham JM (2003). Care for the caregivers: a review of self-report instruments developed to measure the burden, needs, and quality of life of informal caregivers. J Pain Symptom Manag.

[61] Brannan AM, Athay MM, De Andrade ARV (2012). Measurement quality of the caregiver strain questionnaire-short form 7 (CGSQ-SF7). Adm Policy Ment Heal Ment Heal Serv Res.

[62] Bevan JL, Pecchioni LL (2008). Understanding the impact of family caregiver cancer literacy on patient health outcomes. Patient Educ Couns.

[63] Yurk Robin, Morgan David, Franey Steve, Stebner Jennifer Burk, Lansky David (2002). Understanding the Continuum of Palliative Care for Patients and Their Caregivers. Journal of Pain and Symptom Management.

[64] Sleath BL, Carpenter DM, Sayner R, Ayala GX, Williams D, Davis S (2011). Child and caregiver involvement and shared decision-making during asthma pediatric visits. J Asthma.

[65] Graffigna G, Barello S, Libreri C, Bosio C. How to engage type-2 diabetic patients in their own health management: implications for clinical practice. BMC Public Health [Internet]. 2014;14:648. Available from: http://www.biomedcentral.com/1471-2458/14/648 10.1186/1471-2458-14-648 PMC4083034 24966036

[66] Barello S, Graffigna G (2015). Engaging patients to recover life projectuality: an Italian cross-disease framework. Qual Life Res.

[67] Barello S, Graffigna G, Vegni E, Savarese M, Lombardi F, Bosio AC. “Engage me in taking care of my heart”: A grounded theory study on patient- cardiologist relationship in the hospital management of heart failure. BMJ Open [Internet]. 2015;5:1–11. Available from: http://www.embase.com/search/results?subaction=viewrecord%7B&%7Dfrom=export%7B&%7Did=L603571867%5Cn http://dx.doi.org/10.1136/bmjopen-2014-005582%5Cn http://sfxhosted.exlibrisgroup.com/medtronic?sid=EMBASE%7B&%7Dissn=20446055%7B&%7Did=doi:10.1136/bmjopen-20 10.1136/bmjopen-2014-005582 PMC4369000 25776041

[68] Greenhalgh T, Hurwitz B (1999). Narrative based medicine: why study narrative?. BMJ..

[69] Grypdonck MHF (2006). Qualitative health research in the era of evidence-based practice. Qual Health Res.

[70] Smith JAA. Qualitative psychology – a practical guide to research methods. Narrative. 2008;

[71] Mishler EG. Research interviewing: context and narrative. Contemp Sociol 1986.

[72] Robins RW, Fraley CR, Krueger RF. Handbook of research methods in personality psychology. New York. 2007.

[73] Terwee CB, Prinsen CAC, Chiarotto A, Westerman MJ, Patrick DL, Alonso J, et al. COSMIN methodology for evaluating the content validity of patient-reported outcome measures: a Delphi study. Qual Life Res. 2018. 10.1007/s11136-018-1829-0 PMC5891557 29550964

[74] IBM. IBM SPSS Advanced Statistics 22. Ibm. 2013.

[75] Furnham A. Response bias, social desirability and dissimulation. Pers Individ Dif. 1986.

[76] Graffigna G, Barello S, Bonanomi A, Lozza E. Measuring patient engagement: Development and psychometric properties of the patient health engagement (PHE) scale. Front Psychol. 2015;6(MAR). 10.3389/fpsyg.2015.00274 PMC4376060 25870566

[77] Tabachnick BG, Fidell LS. Using multivariate statistics (5th ed.). New York: Allyn and Bacon.

[78] Greco A, Pancani L, Sala M, Annoni AM, Steca P, Paturzo M, et al. Psychometric characteristics of the caregiver burden inventory in caregivers of adults with heart failure. Eur J Cardiovasc Nurs. 2017; 10.1177/1474515117693890 28186426

[79] Marvardi M, Mattioli P, Spazzafumo L, Mastriforti R, Rinaldi P, Polidori MC, et al. The caregiver burden inventory in evaluating the burden of caregivers of elderly demented patients: results from a multicenter study. Aging Clin Exp Res. 2005. 10.1007/BF03337720 15847122

[80] Bandura A. Bandura self-efficacy defined. In: Encyclopedia of Human Behavior. 1994. p. 71–81.

[81] Bonanomi A, Cantaluppi G, Nai Ruscone M, Osmetti SA (2015). A new estimator of Zumbo’s ordinal alpha: a copula approach. Qual Quant.

[82] Bragstad LK, Kirkevold M, Foss C. The indispensable intermediaries: a qualitative study of informal caregivers’ struggle to achieve influence at and after hospital discharge. BMC Health Serv Res. 2014. 10.1186/1472-6963-14-331 PMC4119054 25078610

[83] Kubler-Ross E. On Death and Dying. Vol. 1st, On Death and Dying. 1969. 288 p.

[84] Bonanno GA, Kaltman S (1999). Toward an integrative perspective on bereavement. Psychol Bull.

[85] Hahn-goldberg S, Jeffs L, Troup A, Kubba R, Okrainec K. “We are doing it together ”; the integral role of caregivers in a patients ’ transition home from the medicine unit. PLoS One. 2018:1–14. 10.1371/journal.pone.0197831 PMC5993108 29795623

[86] Dobrof J, Ebenstein H, Dodd S-J, Epstein I. Caregivers and Professionals Partnership Caregiver Resource Center: Assessing a Hospital Support Program for Family Caregivers. J Palliat Med. 2006; 10.1089/jpm.2006.9.196 16430359

[87] Morgan Stephanie, Yoder Linda H. (2011). A Concept Analysis of Person-Centered Care. Journal of Holistic Nursing.

[88] Bell CM, Araki SS, Neumann PJ. The association between caregiver burden and caregiver health-related quality of life in Alzheimer disease. Alzheimer Dis Assoc Disord. 2001; 10.1097/00002093-200107000-00004 11522930

[89] Saunders MM. Influence of heart failure caregiving on caregiver burden, caregiver health-related quality of life and patient hospitalizations. Influence of Heart Failure Caregiving on Caregiver Burden, Caregiver Health-related Quality of Life & Patient Hospitalizations. 2006.

[90] De Valck C, Bensing J, Bruynooghe R, Batenburg V. Cure-oriented versus care-oriented attitudes in medicine. Patient Educ Couns [Internet]. 2001 Nov;45(2):119–126. Available from: http://www.ncbi.nlm.nih.gov/pubmed/11687325. 10.1016/s0738-3991(00)00201-9 11687325

[91] Triberti S, Barello S. The quest for engaging AmI: Patient engagement and experience design tools to promote effective assisted living. J Biomed Inform. 2016:63. 10.1016/j.jbi.2016.08.010 27515924

[92] Graffigna G, Barello S, Bonanomi A, Menichetti J. The motivating function of healthcare professional in eHealth and mHealth interventions for type 2 diabetes patients and the mediating role of Patient engagement. J Diabetes Res. 2016:2016. 10.1155/2016/2974521 PMC4736395 26881243

[93] Retrum JH, Nowels CT, Bekelman DB. Patient and caregiver congruence. J Cardiovasc Nurs 2013; 10.1097/JCN.0b013e3182435f27 22343213

[94] McMullen Carmit K., Schneider Jennifer, Altschuler Andrea, Grant Marcia, Hornbrook Mark C., Liljestrand Petra, Krouse Robert S. (2014). Caregivers as healthcare managers: health management activities, needs, and caregiving relationships for colorectal cancer survivors with ostomies. Supportive Care in Cancer.

[95] Archbold PG, Stewart BJ, Greenlick MR, Harvath T. Mutuality and preparedness as predictors of caregiver role strain. Res Nurs Health. 1990; 10.1002/nur.4770130605 2270302

[96] Rushton CH, Ballard MK. The other side of caring: Caregiver suffering. In: Palliative Care for Infants, Children, and Adolescents : A Practical Handbook. 2011.

[97] Rozario PA, Morrow-Howell N, Hinterlong JE. Role enhancement or role strain: assessing the impact of multiple productive roles on older caregiver well-being. Res Aging. 2004.

[98] Ducharme FC, Lévesque LL, Lachance LM, Kergoat MJ, Legault AJ, Beaudet LM, et al. “Learning to become a family caregiver” efficacy of an intervention program for caregivers following diagnosis of dementia in a relative. Gerontologist. 2011; 10.1093/geront/gnr014 21383112

[99] Applebaum A. Isolated, invisible, and in-need: there should be no i in caregiver. Palliative and Supportive Care 2013. 10.1017/S1478951515000413 PMC4980827 25994477

[100] Gilbar O. Parent caregiver adjustment to cancer of an adult child. J Psychosom Res. 2002. 10.1016/s0022-3999(01)00259-8 12023126

[101] Gilley DW, McCann JJ, Bienias JL, Evans DA. Caregiver psychological adjustment and institutionalization of persons with Alzheimer’s disease. J Aging Health. 2005; 10.1177/0898264304274252 15750050

[102] Peterson KJ (1985). Psychosocial adjustment of the family caregiver: home hemodialysis as an example. Social Work Health Care.

[103] Kim Y, Baker F, Spillers RL, Wellisch DK (2006). Psychological adjustment of cancer caregivers with multiple roles. Psychooncology.

[104] Graffigna G, Barello S, Bonanomi A. The role of Patient Health Engagement model (PHE-model) in affecting patient activation and medication adherence: A structural equation model. PLoS One. 2017;12(6). 10.1371/journal.pone.017986. 10.1371/journal.pone.0179865 PMC5487073 28654686

[105] Graffigna G, Barello S, Riva G, Savarese M, Menichetti J, Castelnuovo G, et al. Fertilizing a patient engagement ecosystem to innovate healthcare: Toward the first Italian Consensus conference on patient engagement. Front Psychol. 2017;8(JUN). 10.3389/fpsyg.2017.00812 PMC5460315 28634455

[106] Stephens MAP, Townsend AL, Martire LM, Druley JA. Balancing Parent Care With Other Roles: Interrole Conflict of Adult Daughter Caregivers. Journals Gerontol Ser B Psychol Sci Soc Sci. 2001; 10.1093/geronb/56.1.p24 11192334

[107] Guida E, Barello S, Corsaro A, Galizi MC, Giuffrida F, Graffigna G, et al. An Italian pilot study of a psycho-social intervention to support family caregivers ’ engagement in taking care of patients with complex care needs : the engage-in-caring project. BMC Health Serv Res. 2019:1–8. 10.1186/s12913-019-4365-x PMC6679432 31375099

